# Beyond the bugs: why toxin detection is key in two-tiered *C. diff* tests

**DOI:** 10.1017/ash.2025.10196

**Published:** 2025-10-24

**Authors:** David Goldmeier, Michael Parry, Marina Mirkovic, Asha Shah, Shweta Karki, Forugh Homayoonrooz, Suzanne J. Rose

**Affiliations:** 1 George Washington University Hospital, Washington, DC, USA; 2 Stamford Hospital, Department of Medicine, Stamford, CT, USA; 3 Stamford Health Medical Group, Stamford, CT, USA; 4 Stamford Health Department of Research and Discovery, Stamford, CT, USA

## Abstract

**Objective:**

We recently added *C. diff* toxin assay to polymerase chain reaction (PCR) as a two-tier algorithm to investigate whether this approach improves patient outcomes and antibiotic stewardship.

**Design:**

Retrospective chart review.

**Setting:**

305-bed acute care urban teaching hospital.

**Patients:**

All inpatients admitted who tested positive for *C. diff* by PCR. Testing was performed by provider order on clinical suspicion of infection. Exclusion criteria were patients with chronic diarrhea, history of IBD, and recent gastric bypass surgery.

**Methods:**

On April 1, 2023, the two-tier testing algorithm was implemented for patients who tested positive by PCR. The EMR was reviewed through May 1, 2024, to determine whether toxin-positive patients differed from toxin-negative patients with respect to their demographics, clinical characteristics, outcomes, and initiation of antibiotic treatment.

**Results:**

Of 147 consecutive *C. diff* PCR-positive patients, 32% tested toxin-positive (*n* = 57) and 51% toxin-negative (*n* = 90). Demographics were similar across groups. Toxin-positive patients showed more symptoms of colitis, more bloating, a higher average white blood cell count, and had a higher fatality rate. Antibiotics were more commonly prescribed to toxin-positive patients (98%) than toxin-negative patients (56%) (*p* < 0.01). Of the 90 patients who were toxin-negative, 44% of those were not treated with antibiotics.

**Conclusion:**

Our study supports previous findings that a two-tier testing strategy effectively identifies active *C. diff* infection rather than colonization, effectively improving antibiotic stewardship efforts. Some toxin-negative patients also had colitis symptoms and responded to antibiotics, indicating that clinical judgment is still needed in cases with discrepant results.

## Introduction

Despite advances in the detection, treatment, and prevention of *Clostridiodes difficile* (*C. diff*) infections, they remain prevalent in the community and are the leading cause of hospital-acquired infection in the United States.^1^ Polymerase chain reaction (PCR) has been the most commonly used method for detecting *C. diff* in stool samples, but it has become increasingly apparent that this single test is unable to differentiate between colonization and active infection with *C. diff*. Older methods using enzyme-linked immunoassays were less sensitive than PCR but had the advantage of detecting toxin A and/or B production which has been shown to correlate better with *C. diff* colitis.^2^ As a result, clinicians using only PCR for diagnosis often fail to distinguish between colonization and infection and their institutions report higher rates of *C. diff* and higher rates of *C. diff* treatment.

Most facilities now use a combination of tests to diagnose clinically significant *C. diff* infection.^3^ Studies using a 2-step method (PCR followed by toxin testing), show lower rates of *C. diff* and improved antibiotic stewardship by reducing antibiotic treatment that is often considered unnecessary in colonized patients.^4–6^ Recently, Stamford Hospital added *C. diff* toxin assay as a reflex test following a positive PCR to help identify true cases of infection, while discouraging treatment of patients who are only colonized. We prospectively investigated whether the inclusion of this additional layer of testing impacted patient outcomes and led to better antibiotic stewardship.

### Study design and methods

Prior to study initiation, approval was received by the Institutional Review Board (WCG IRB Work Order #1–1 736 259–1). We performed a retrospective review of inpatients admitted to Stamford Hospital who tested positive for *C. diff* by PCR (Cepheid GeneXpert Systems, Sunnyvale California) between November 1, 2023, and May 1, 2024. Stamford Hospital is a 305-bed acute care urban teaching hospital in Connecticut with a primary and secondary service area totaling approximately 250,000 people. *C. diff* testing was performed by provider order on clinical suspicion and required provider’s written certification that the patient have at least three watery bowel movements within 24 hours, and the diarrhea was not due to cathartics, oral contrast, or tube feedings. Additionally, clinical suspicion of colitis was required (ie, leukocytosis, fever, lower abdominal pain, and cramping). Exclusion criteria were patients with chronic diarrhea (at least 4 wk duration of symptoms), history of IBD (Crohn’s disease, ulcerative colitis, microscopic colitis), and recent gastric bypass surgery.

Prior to April 1, 2023, the results of *C. diff* PCR testing were reported as positive or negative and no toxin resting was performed resulting in essentially all positive cases treated with antibiotics. After April 1, 2023, toxin testing (*C. diff* Quik Chek, Techlab, Blacksburg, VA) was reflexively added as an additional diagnostic layer and formal recommendations were made to clinicians that patients with a negative toxin test result represent colonization rather than infection and may not need to be treated.

Patient demographic information, including race, sex, age was collected for all patients meeting inclusion criteria from the hospital’s electronic medical records. Additional clinical data, such as leukocyte counts, erythrocyte sedimentation rate (ESR), and C-reactive protein (CRP) were also captured. The presence of clinical features such as abdominal pain, abdominal pain score, bloating, symptoms of colitis, antibiotic treatment for *C. diff*, and fatality were determined by medical record review.

### Statistical analysis

Statistical analyses were conducted using SAS version 9. χ^2^ tests compared discrete variables, t-tests assessed continuous variables, and Mann–Whitney U tests were used for small sample sizes. A *P* -value <.05 was deemed statistically significant. No imputation was used for missing data.

## Results

A total of 147 patients met inclusion criteria for analysis of which, 57 were toxin-positive (32%) and 90 were toxin-negative (51%). Table 1 shows the overall demographics including race, sex, and age among toxin results group. There were no significant differences in demographic characteristics among the cohorts with respect to race, gender, or age.

**Table 1.** Overall demographics of PCR positive based on *C. diff* toxin results

Variable	Category	*C. diff* toxin result		p-value
		**Positive (*n* = 57)**	**Negative (*n* = 90)**	
**Race**	White Black Mixed Asian Hispanic Unknown	38 (66.67%)4 (7.03%)3(5.26%)4 (7.03%)4 (7.03%)4 (7.03%)	54 (60%)7 (7.78%)7 (7.78%)4(4.44%)7 (7.78%)11 (12.22%)	0.85
**Sex**	Female Male	30 (52.63%) 27 (47.37%)	43 (47.78%) 47 (52.22%)	0.56
**Age**	Years Mean (SD)	71.38 (16.22)	67.13 (17.99)	0.41

PCR, polymerase chain reaction; c.diff, Clostridiodes difficile; SD, standard deviation.

Table 2 shows the distribution of clinical variables between patients with a positive compared with a negative toxin result. 82% of patients with a positive result toxin had symptoms of colitis compared to 38% of those with a negative toxin result, a statistically significant finding (*P* < .01). Similarly, a significant difference was found in the white blood cell count (WBC) count: toxin-positive patients had a higher mean WBC count of 15,000 cells/mcl while toxin-negative patients had a WBC count of 11,000 cells/mcl (*P* < .01). There were no differences between the cohorts in CRP or ESR values. Symptoms of bloating were more common in toxin-positive (48%) compared with toxin-negative (31%) patients (*P* = .04) but there was no difference in initial or final abdominal pain scores. 98% of patients who had a positive toxin were treated with antibiotics while those with a negative toxin result were significantly less likely to be treated, 56% (*P* < .01). There was also a significant difference in a fatal outcome by *C. diff* toxin result: 12 patients in the toxin-positive group expired compared with 4 patients in the toxin negative group (*P* < .01).

**Table 2.** Clinical comparison among positive vs negative toxin result

Variable	Category	*C. diff* toxin result		P-value
		**Positive (*n* = 57)**	**Negative (*n* = 90)**	
**Initial abdominal pain score**	0–10	2.84 (3.57)	2.60 (3.39)	0.68
**Final abdominal pain score**	0–10	0.80 (1.92)	0.70 (1.78)	0.73
**Symptoms of colitis**	YesNo	47 (82.45%)10 (17.54%)	34 (37.78%)56 (62.22%)	<0.01
**WBC**	Mean (SD)	14.79 (8.44)	10.79 (5.0)	<0.01
**CRP**	Mean (SD)	119.64 (71.56)	63.57 (56.20)	0.15*
**ESR**	Mean (SD)	49.0 (45.32)	72.13 (67.78)	0.59*
**Abdominal pain**	YesNo	34 (59.65%)23 (40.35%)	43 (47.78%)47 (52.22%)	0.16
**Bloating**	YesNo	27 (48.21%)29 (51.79%)	28 (31.11%)62 (68.89%)	0.04
**Antibiotic treatment for *C. diff***	YesNo	56 (98.25%)1 (1.75%)	50 (55.56%)40 (44.44%)	<0.01
**Death from *C. diff***	YesNo	12 (21.05%)45 (78.95%)	4 (4.44%)86 (95.56%)	<0.01.

c.diff, Clostridiodes difficile; SD, standard deviation; WBC, white blood cell; CRP, C-reactive protein; ESR, erythrocyte sedimentation rate. * Mann–Whitney U tests were used for small sample sizes.

A sub-analysis was conducted for clinical variables among positive and negative toxin results among those who had symptoms of colitis (*n* = 81). 34 of these patients (38%) were toxin-negative and 47 (46%) were toxin-positive. Most of the toxin-positive patients (98%) were treated with antibiotic compared to 65% of those who were toxin-negative (*P* < .01) (Table 3). The average initial pain score among toxin-negative patients was 3.63 and the average final pain score was lowered significantly to 1.09 (*P* < .01), indicating symptomatic improvement. Symptomatic improvement was also found among toxin-positive patients with average initial pain score of 3.38 and was lowered to .89 (*P* < .01) (Table 3).

**Table 3.** Sub-analysis of patients who had symptoms of colitis among positive vs negative toxin result (*n* = 81)

Variable	Category	C diff toxin result		p-value
		**Positive (*n* = 47)**	**Negative (*n* = 34)**	
**Antibiotic**	YesNo	46 (97.87%)1 (2.13%)	22 (64.71%)12 (35.29%)	<0.0001

C.diff, *Clostridiodes difficile*; SD, standard deviation.

## Discussion

This study demonstrates the efficacy of a two-tier testing strategy for *C. diff*, utilizing a sensitive screening test followed by a confirmatory toxin assay, in accurately discriminating between true infection and asymptomatic colonization. The implementation of this strategy resulted in a significant improvement in antibiotic stewardship, specifically a reduction in inappropriate treatment for individuals without clinically significant *C. diff* infection.

Our study confirms the results of others,^7^ that implementation of a two-tier testing strategy can help discriminate between “true” *C. diff* infections (PCR+/Toxin+) and colonization (PCR+/Toxin-). Symptoms of colitis were present in 82% of patients who were toxin-positive and 98% needed antibiotic treatment. Furthermore, these true cases were associated with a statistically higher number of *C. diff* fatalities compared to patients who were colonized (toxin-negative). However, some toxin-negative patients had symptoms of colitis (38%) accompanied by abdominal pain and elevated ESR and they responded to antibiotics. Therefore the two-tier testing strategy is not infallible and should not be used to deny treatment. A recent survey of physicians indicated that regardless of clinical background and experience, on-going challenges continue to be presented in the treatment of C.diff^8^ and therefore clinical judgment cannot be underestimated when treating this patient population.

The observed improvement in antibiotic stewardship is a crucial finding of this study. The reduction in unnecessary antibiotic use aligns with established guidelines emphasizing the importance of targeted therapy for symptomatic C.diff infections^9^ while avoiding treatment for asymptomatic carriers. This practice not only minimizes the risk of adverse drug reactions and the development of antibiotic resistance but also reduces healthcare costs associated with prolonged and inappropriate antibiotic therapy. In our study, toxin-negative patients did not need, or receive, antibiotic treatment in over 40% of cases. This validated our educational efforts during the transition to the two-tier process and the effectiveness of our order entry “pop-up” reminders. Additionally, following initiation of toxin testing, hospitalwide memos were shared, and staff members were educated through presentations at grand rounds, house staff and hospitalist meetings, nursing and laboratory staff were also included as well.

Several limitations should be considered when interpreting our results. Firstly, the study was conducted at a single institution, potentially limiting the generalizability of our findings to other healthcare settings, although we did include all inpatient *C. diff* cases over the six-month study period. Secondly, the retrospective nature of the analysis may have introduced biases, such as selection bias or incomplete data capture. Thirdly, inflammatory markers, such as ESR and CRP, were not ordered consistently enough to provide more support in diagnosing colitis. Finally, many patients in this study had a history of recurrent *C. diff* infections but this was not included as a variable in the study so the impact of this history on our results is unknown.

The implementation of a two-tier testing strategy for *C. diff* significantly improved the accuracy of C.diff infection diagnosis and promoted antibiotic stewardship. By effectively differentiating between true C.diff infections and colonization, this approach reduced the unnecessary use of targeted antibiotics, contributing to better patient outcomes and mitigating the risks associated with antimicrobial resistance at our community teaching hospital. Future research should focus on validating these findings in diverse clinical settings and optimizing the selection of screening and confirmatory assays for optimal diagnostic accuracy and clinical impact.

## References

[1] Mada PK , Alam MU. *Clostridioides difficile* infection. In: StatPearls. StatPearls Publishing; 2025. Accessed March 21, 2025. 28613708

[2] Cymbal M , Chatterjee A , Baggott B , Auron M. Management of *Clostridioides difficile* infection: diagnosis, treatment, and future perspectives. Am J Med 2024;137:571–576. 38508330 10.1016/j.amjmed.2024.03.024

[3] Carroll KC , Mizusawa M. Laboratory tests for the diagnosis of *Clostridium difficile* . Clinics in Colon and Rectal Surg 2020;33:073–081. 10.1055/s-0039-3400476 PMC7042017 32104159

[4] Hitchcock MM , Holubar M , Hogan CA , Tompkins LS , Banaei N. Dual Reporting of C*lostridioides difficile* PCR and Predicted Toxin Result Based on PCR Cycle Threshold Reduces Treatment of Toxin-Negative Patients without Increases in Adverse Outcomes. Tang YW, ed. J Clin Microbiol 2019;57:e01288–19. 31511334 10.1128/JCM.01288-19 PMC6812995

[5] Dbeibo L , Lucky CW , Fadel WF , et al. Two-step algorithm-based *Clostridioides difficile* testing as a tool for antibiotic stewardship. Clin Microbiol Infect 2023;29:798.e1–798.e4. 10.1016/j.cmi.2023.02.008 36804907

[6] Bettger CC , Giancola SE , Cybulski RJ , Okulicz JF , Barsoumian AE. Evaluation of a two step testing algorithm to improve diagnostic accuracy and stewardship of *Clostridioides difficile* infections. BMC Res Notes 2023;16:172. 37580824 10.1186/s13104-023-06398-9 PMC10426052

[7] Turner NA , Krishnan J , Nelson A , et al. Assessing the impact of 2-step *Clostridioides difficile* testing at the healthcare facility level. Clin Infect Dis 2023;77:1043–1049. 37279965 10.1093/cid/ciad334 PMC10552580

[8] Nath SG , Lee F , Bararia A , Nijhawan AE. 781. *C.difficile* PCR+/ toxin EIA- treat or not treat? A clinician survey. Open Forum Infect Dis 2020;7:S435–S436.

[9] Johnson S , Lavergne V , Skinner AM , et al. Clinical Practice Guideline by the Infectious Diseases Society of America (IDSA) and Society for Healthcare Epidemiology of America (SHEA): 2021 focused update guidelines on management of *Clostridioides difficile* infection in adults. Clin Infect Dis 2021;73:e1029–e1044. 34164674 10.1093/cid/ciab549

